# Without safeguards, AI-Biology integration risks accelerating future pandemics

**DOI:** 10.3389/fmicb.2025.1734561

**Published:** 2026-01-22

**Authors:** Dianzhuo Wang, Marian Huot, Zechen Zhang, Kaiyi Jiang, Eugene I. Shakhnovich, Kevin M. Esvelt

**Affiliations:** 1 Department of Chemistry and Chemical Biology, Harvard University, Cambridge, MA, United States; 2 Laboratory of Physics, Ecole Normale Supérieure and PSL Research, Paris, France; 3 Department of Physics and Center for Brain Science, Harvard University, Cambridge, MA, United States; 4 Omenn-Darling Bioengineering Institute, Princeton University, Princeton, NJ, United States; 5 Media Lab, Massachusetts Institute of Technology, Cambridge, MA, United States

**Keywords:** protein language models, intelligent automated biology, dual use research of concern (DURC), biosecurity, protein design

## Abstract

Artificial intelligence now shapes the design of biological matter. Protein language models (pLMs), trained on millions of natural sequences, can predict, generate, and optimize functional proteins with minimal human input. When embedded in experimental pipelines, these systems enable closed-loop biological design at unprecedented speed. The same convergence that accelerates vaccine and therapeutic discovery, however, also creates new dual-use risks. We first map recent progress in using pLMs for fitness optimization across proteins, then critically assess how these approaches have been applied to viral evolution and how they intersect with laboratory workflows, including active learning and automation. Building on this analysis, we outline a capability-oriented framework for integrated AI–biology systems, identify evaluation challenges specific to biological outputs, and propose research directions for training- and inference-time safeguards.

## 1 Introduction: the new biosecurity frontier in AI

Artificial intelligence (AI) is transforming the practice of biological discovery. Among the most powerful tools driving this change are protein language models (pLMs)—large models trained on vast collections of natural protein sequences (Xiao et al., 2025). These tools offer unprecedented speed and scope for understanding biological systems, predicting properties like protein fitness (Chen et al., 2023; Meier et al., 2021; Vieira et al., 2024; Zhang et al., 2024), and even generating novel proteins entirely (Ruffolo and Madani, 2024; Hsu et al., 2022). In the recent fight against SARS-CoV-2 pandemic, pLMs help predicting viral fitness (Wang et al., 2024; Yu et al., 2025; Ito et al., 2024) and immune escape (Wang et al., 2023), accelerate the development of vaccines and therapeutics (Hie et al., 2024; Jiang et al., 2024; Shanker et al., 2024) and anticipate viral evolution (Huot et al., 2025b,c).

However, the true transformative power, and potential risks, emerge not from pLMs in isolation, but from their integration with wet lab platforms and active learning close feedback loops. This convergence, which we term *Intelligent Automated Biology* (IAB), couples model-guided sequence design with robotic synthesis and experimental feedback, creating a high-throughput loop that iteratively refines biological function. Such systems promise major advances in therapeutic discovery, enzyme design, and pandemic preparedness. Yet they also reshape the landscape of biosecurity by enabling optimization of viral traits or other high-risk functions with diminishing human oversight.

Rather than portraying IAB as a singular threat, our goal is to examine how its technical trajectory alters the biosecurity framework. The integration of predictive modeling, active learning, and automated experimentation yields three interlocking effects. First, the exploration of protein fitness landscapes is dramatically accelerated: active learning allows the efficient identification of functional mutations from minimal data (Jiang et al., 2024; Huot et al., 2025b; Yang et al., 2025). Second, laboratory throughput expands by orders of magnitude, with automated platforms capable of testing thousands of variants per day (Zhang et al., 2025; Yu et al., 2023). Finally, these tools collectively lower the expertise required to perform sophisticated protein engineering, widening access to capabilities once restricted to specialized laboratories.

In this Perspective, we map the current progress in fitness optimization with pLMs and critically assess how these approaches are being applied to viral evolution and integrated with automated laboratory workflows. We then propose a capability-oriented framework for evaluating integrated AI–biology systems and outline emerging directions for pLM-specific safeguards.

## 2 Protein language models: a leap in prediction capability

### 2.1 Backgrounds

Inspired by advances in natural language processing, pLMs are trained on large databases of unaligned natural protein sequences using self-supervised objectives. In the *autoregressive* setting, a pLM is trained to predict the next amino acid in a sequence, modeling the joint probability of a protein sequence *x* = (*x*_1_, *x*_2_, …, *x*_*L*_) as:


$$\begin{array}{l} P\left(x\right)=\overset{L}{\underset{i=1}{\prod }}P\left(x_{i}|x_{1},\ldots ,x_{i-1}\right), \end{array}$$ (1)

capturing sequential and context-dependent dependencies across residues. This setup is particularly suited for sequence generation and allows scoring of full sequences or specific mutations via log-likelihood comparisons.

A key advantage of pLMs is that they operate directly on raw sequence data, eliminating the need for the time-consuming and often difficult step of creating multiple sequence alignments required by earlier methods. This makes pLMs more flexible and substantially faster to deploy, especially for novel proteins or viruses where alignment data is limited.

**Figure 1:**
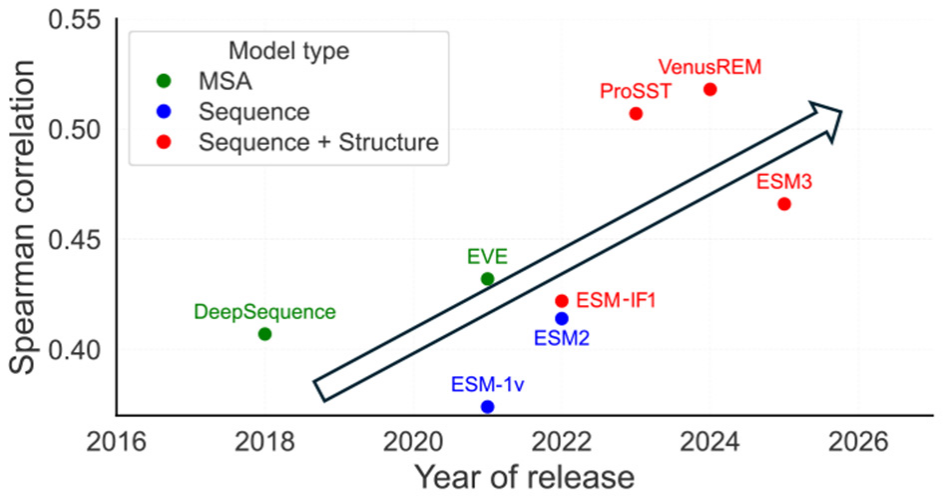
Model performance improves over time. Spearman correlation coefficients between predicted mutational effects and experimental ground truth on ProteinGym, colored by input type: MSA-based (green) (Riesselman et al., 2018; Frazer et al., 2021), sequence-based (blue) (Meier et al., 2021; Lin et al., 2022), or structure + sequence based (red) (Li et al., 2024; Hsu et al., 2022; Hayes et al., 2024).

Furthermore, because pLMs are trained on such vast and diverse datasets, they learn highly general principles of protein biology. This allows a single, large pre-trained pLM—such as those in the widely used ESM family (Meier et al., 2021; Lin et al., 2022)—to make meaningful predictions about virtually any protein sequence, even those belonging to protein families not seen during training. This capability is known as “zero-shot” prediction. Recent advances have further improved the performance of pLMs by explicitly incorporating structural information into the model (Figure 1). For example, ESM-3 (Hayes et al., 2024) unifies sequence and structure modeling by co-training across multiple modalities, including 3D coordinates, sequence likelihood, and masked token recovery. This joint training enables improved accuracy in predicting mutational effects and sequence plausibility within structural constraints. Additionally, some inverse folding models, like ESM-IF (Hsu et al., 2022), and ProteinMPNN (Dauparas et al., 2022) are structure-conditioned; they can predict sequences likely to fold into a specific 3D shape, or assess how well a mutation fits within a known structure. Beyond pLMs, other architectures offer similar capabilities. FAMPNN (Shuai et al., 2025) extends ProteinMPNN (Dauparas et al., 2022) by jointly modeling sequence identity and sidechain structure through combined masked language modeling and coordinate denoising. A distinct class of models focuses on *de novo* backbone generation: RFdiffusion (Watson et al., 2023) employs diffusion processes to construct novel protein structures from noise, enabling both unconditional topology design and conditional generation with explicit constraints (e.g., scaffolding functional motifs or designing target binders). While these structure-generation models differ architecturally from pLMs, they demonstrate comparable capabilities in rational protein design.

### 2.2 Models for viral protein properties prediction

(Hie et al. 2021) demonstrated that pLMs, when trained solely on viral sequence data without fine-tuning or structural supervision, can capture both the functional and antigenic consequences of mutations. They trained separate BiLSTM language models on corpora of aligned sequences for influenza HA, HIV Env, and SARS-CoV-2 Spike, and introduced the Constrained Semantic Change Search (CSCS) framework. In this framework, grammaticality (i.e., the model-assigned likelihood of a sequence) was hypothesized to reflect viral fitness, while semantic change (i.e., the shift in embedding space) served as a proxy for immune escape. Despite being trained only on viral sequences and without escape labels, the models successfully predicted known escape mutations in a zero-shot setting, highlighting the capacity of language models to learn biologically meaningful patterns directly from sequence data.

Building on this, (Allman et al. 2024) systematically benchmarked grammaticality and semantic change across multiple viral proteins using both the original LSTM-based model and newer pretrained pLMs like ESM-2. Their analysis confirmed that grammaticality scores are consistently higher for viable mutations and can serve as a practical proxy for fitness. This finding held across viral systems, including HIV and influenza. In parallel, (Wang et al. 2024) used ESM embeddings to predict the fitness of SARS-CoV-2 RBD variants by integrating them into a biophysical model. More broadly, other pLMs and AI models have been developed to predict key viral properties such as binding affinity (Wang et al., 2023; Loux et al., 2024; Taft et al., 2022; Basse et al., 2025; Liu et al., 2023), mutation spread (Maher et al., 2022), and fitness (Ito et al., 2024; Zhang et al., 2024).

Collectively, these results underscore a crucial point: powerful pLMs, including those trained broadly rather than exclusively on viral data, encode meaningful information about viral protein function and evolution. This enables them to anticipate evolutionary trajectories and assess mutational effects in emerging pathogens, often with remarkable accuracy directly from sequence data.

Importantly, while these models were developed to support beneficial applications like vaccine design or pandemic forecasting, their predictive capabilities could also be misused. Moreover, because many pLMs are open-weight and require minimal fine-tuning, such capabilities may be accessible even without deep virological expertise. **Notably, these tools have been used to design novel SARS-CoV-2 proteins that were experimentally shown to be infectious and capable of evading neutralization** (Youssef et al., 2025; Huot et al., 2025a).

## 3 The accelerator effect: integrating AI with lab experiments

pLMs are not just predictive tools; they are increasingly integrated into active protein engineering workflows, dramatically accelerating the pace and changing the nature of biological design. This integration manifests in several key applications.

### 3.1 Smarter directed evolution

Directed evolution is a laboratory technique that mimics natural selection to improve proteins for specific purposes, such as improving the efficiency of enzymes, increasing binding affinity of therapeutic antibodies (Jiang et al., 2024). Traditionally, this involves creating large libraries of protein variants and screening them for desired properties, often a laborious, inefficient, and expensive process. **pLMs enables the direct evolution of novel proteins with substantially improved functional properties**. By predicting the likely effects of mutations by either zero shot or few shot, pLMs can guide researchers to focus on variants with a higher probability of success, effectively narrowing down the search space and reducing the experimental burden. Recent studies have demonstrated that general and structure-informed pLMs can substantially improve the binding affinity and neutralization breadth of human antibodies against diverse viral targets, including SARS-CoV-2, Ebola, and influenza, while requiring only minimal rounds of experimental screening (Hie et al., 2024; Shanker et al., 2024; Shan et al., 2022).

### 3.2 Laboratory automation and closed-loop experimentation

**Figure 2:**
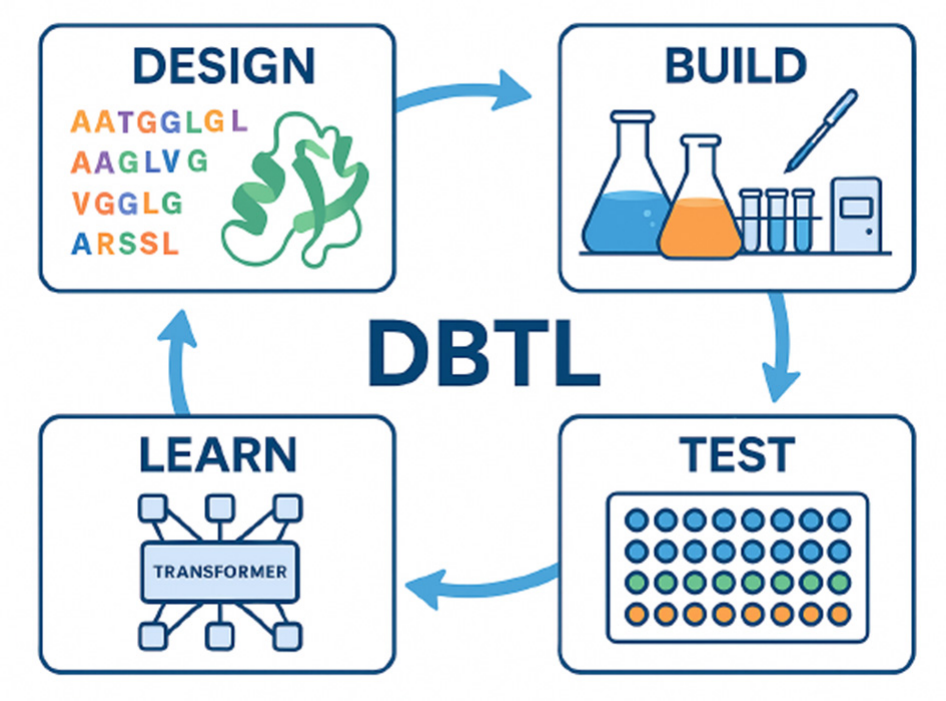
Schematic of the DBTL cycle in AI-enabled bioengineering. pLMs propose novel sequences (Design), which are synthesized and expressed by robotic platforms (Build), evaluated through high-throughput assays (Test), and iteratively improved based on experimental feedback (Learn).

The impact of pLMs is amplified when combined with laboratory automation, often referred to as “biofoundries” (Hillson et al., 2019; Torres-Acosta et al., 2022). This integration enables fully automated cycles of biological design, construction, testing, and learning (Figure 2), commonly known as the Design–Build–Test–Learn (DBTL) cycle. The DBTL cycle includes: (1) Design: AI/pLMs propose sequences with predicted properties; (2) Build: Robotic systems synthesize DNA and produce variants; (3) Test: Automated assays measure properties; (4) Learn: Results feed back to AI for improved designs in subsequent cycles.

Platforms like PLMeAE (Zhang et al., 2025) demonstrate the power of this approach, achieving multiple rounds of enzyme optimization in just 10 days—a task that could take many months using traditional methods (Zhang et al., 2025). This creates a powerful, high-speed, closed loop for biological engineering. While offering tremendous potential for accelerating therapeutic development, this automation also raises concerns. The speed and reduced human intervention inherent in these closed loops could potentially allow for the rapid optimization of harmful properties if misused, with fewer opportunities for oversight or ethical review during the process.

### 3.3 Efficient exploration with active learning

The sheer number of possible mutations, even within a single protein, makes exhaustive experimental or computational testing infeasible. Active learning offers a solution by integrating model predictions with experimental design (Yang et al., 2025; Balashova et al., 2025). Instead of testing randomly or relying solely on initial predictions, active learning uses the predictive models to select the most informative experiments to perform at each stage, based on certain acquisition function (Margatina et al., 2021).

The typical process starts with wet-lab testing a small, initial set of variants. The results are used to train or fine-tune a predictive model (like a pLM) (Schmirler et al., 2023). The model then evaluates the vast pool of untested variants and identifies those whose experimental evaluation would maximally improve the model's accuracy or are most likely to possess the desired properties (e.g., high fitness, activity, or strong binding). These selected variants are then synthesized and tested, and the new data is used to update the model, repeating the cycle. This iterative strategy dramatically reduces the number of experiments required to explore the mutational landscape and identify top-performing or high-risk variants. Active learning has already shown success in domains like drug discovery (Fralish and Reker, 2024; Graff et al., 2021; Warmuth et al., 2001; Bailey et al., 2023).

Recent studies have shown that active learning frameworks can optimize enzymes, antibodies, or other protein variants, antibody or protein variants substantially faster than random screening, using only a small fraction of what traditional method required (Jiang et al., 2024; Yang et al., 2025). This efficiency can also enable researchers to proactively identify concerning viral mutations before they potentially emerge naturally (Huot et al., 2025b).

**The synergy between pLMs (for design and prediction), active learning (for efficient experimental guidance), and laboratory automation (for rapid build and test cycles) creates an engineering capability greater than the sum of its parts**. This integrated approach enables systematic biological exploration and optimization at an unprecedented speed and scale. While this accelerates beneficial research, it simultaneously increases the risk of malicious biological engineering and potentially reduces human oversight within automated loops.

## 4 The dual-use dilemma: assessing risks of IAB

The core challenge presented by the convergence of AI and biotechnology lies in its inherent dual-use nature: technologies developed with beneficial intent, such as improving human health or combating pandemics, can often be repurposed to cause harm. pLM substantially amplifies this dilemma by accelerating design cycles, lowering knowledge barriers, and enabling automation at unprecedented scales. To effectively discuss and manage these risks, it is helpful to categorize the capabilities enabled by IAB and assess their associated risk levels.

We propose a framework categorizing IAB capabilities into five levels, reflecting escalating potential for misuse as pLM integration deepens (Table 1). This framework builds upon initial concepts and incorporates insights from recent literature on AI capabilities and biosecurity risks.

**Table 1:** IAB capability levels and associated biosecurity risk.

**Capability level**	**Description**	**Examples**	**Base risk level**
Level 1: Zero-shot prediction	basic pLM predictions (e.g., sequence likelihood as fitness proxy).	ESM-1v zero-shot prediction with grammaticality score (Meier et al., 2021; Allman et al., 2024).	Low–Moderate
Level 2: Advanced prediction & analysis	Accurate ML/pLM prediction of complex molecular properties (e.g., binding affinity changes (Δ*ΔG*), immune escape potential, stability).	Fine-tuned ESM3 to predict viral fitness; UniBind (Wang et al., 2023) predicting binding affinity; EVEscape (Thadani et al., 2023) and VIRAL (Huot et al., 2025b) predicting escape variants; MMSite for active site prediction (Ouyang et al., 2024)	Moderate
Level 3: Targeted sequence generation	Generative AI/pLMs designing novel sequences optimized for specific functional properties (e.g., enhanced binding, stability, potentially virulence factors or toxins).	ProteinMPNN (Dauparas et al., 2022) or ESM-IF1 (Hsu et al., 2022) for generative enzyme/antibody design; Potential toxin/pathogen design.	High
Level 4: Integrated design & active learning	Combining generative models with active learning/Bayesian optimization for efficient discovery and optimization of desired (potentially harmful) biological functions.	ProteinNPT (Notin et al., 2023) for Active learning frameworks; EVOLVEpro (Jiang et al., 2024) and ALDE (Yang et al., 2025) for direct evolution;	Very High
Level 5: Full AI-Bio automation integration	Closed-loop systems linking AI protein design, learning, synthesis, and testing (DBTL cycle) with minimal human oversight	PLMeAE (Zhang et al., 2025) or iBioFAB (Yu et al., 2023) where pLMs are embedded in automated biofoundries	Extremely High

This table illustrates that while Level 2 already poses a threat by enabling designing viral proteins that escape antibody binding, the most substantial risks emerge from the DBTL cycle coupled with physical automation. Level 5 enabling rapid, automated, and potentially remote execution of complex bioengineering tasks,^1^ maximizing both the potential for benefit and the potential for misuse. For each level we classified, concrete examples are provided—and concerningly, full AI-biology automation integration at Level 5 has already been observed in 2025. A key rationale for this tiered framework is that it enables the development of proportionate safeguards tailored to each IAB capability level.The specific design and implementation of such tiered safeguards, while outside the scope of this work, represents a critical direction for future policy development and technical research for IAB.

**Figure 3:**
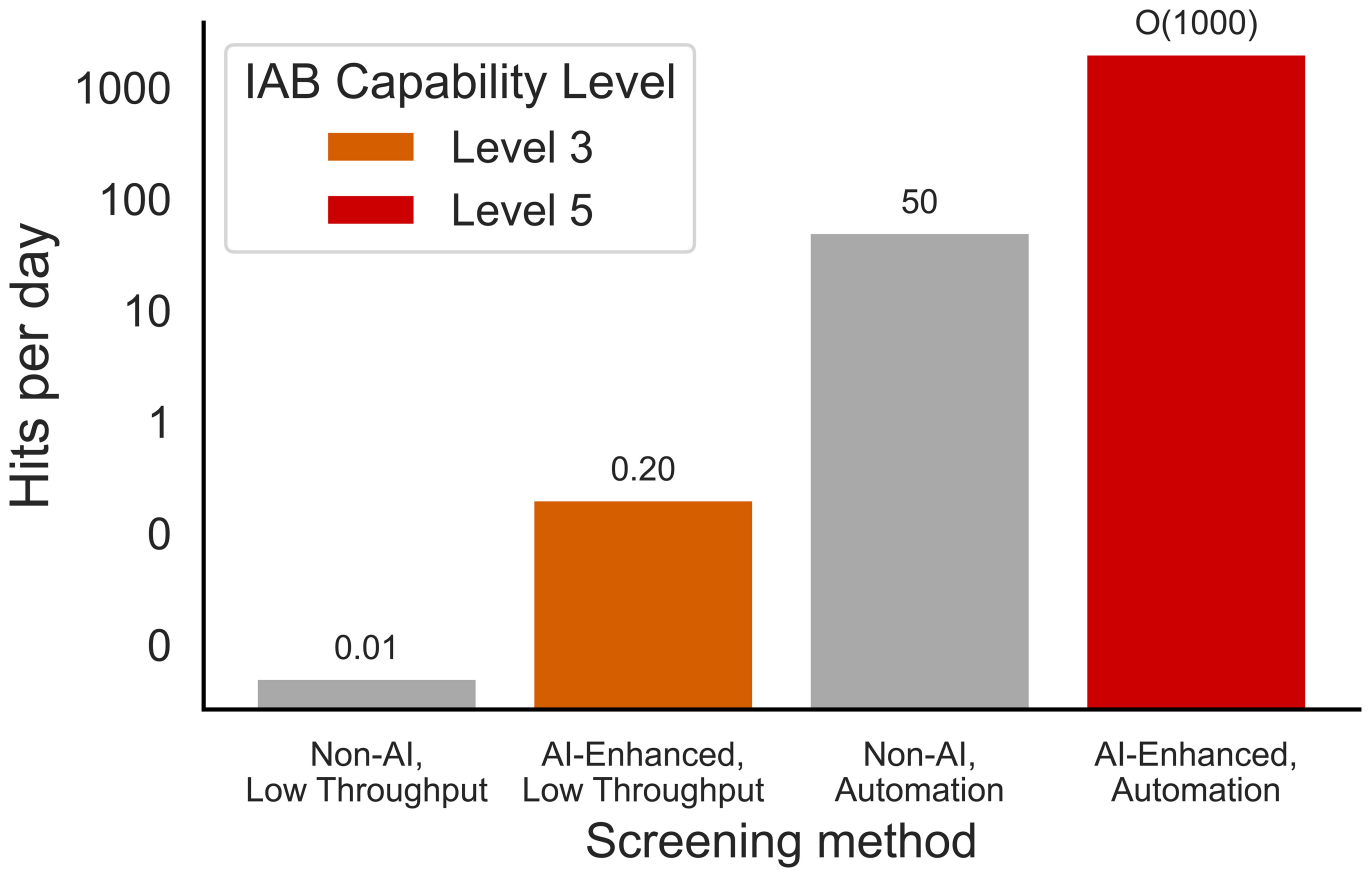
Functional protein “hits” per day from AI vs non-AI methods under low- and high-throughput settings. Based on throughput x hit rate estimated from study on a 85 amino acid protein (Jawaid et al., 2023).

To better quantify the acceleration enabled by this integration, we estimated the speed to obtain a functional variant (“hit”) using wet-lab hit rates on an 85-amino-acid peptide (Jawaid et al., 2023). Hit rate is defined as the fraction of tested sequences that exhibit the desired function. Combining these hit rates with representative experimental throughput values, we find that AI-guided, automated pipelines (Level 5) can yield thousands of hits per day—several orders of magnitude more than traditional, manual, non-AI-guided approaches (Figure 3). This illustrates how full-stack automation not only increases capability but compresses timelines, potentially outpacing the safety checks traditionally used to govern wet-lab experimentation.

A critical factor contributing to this assessment difficulty is the “evaluation bottleneck” (Pannu et al., 2025). AI-Bio models at Capability Level 3 and above can generate novel protein sequences, but accurately predicting their real-world function—especially their potential harmfulness—remains an open challenge. Definitive functional validation often requires synthesizing the DNA and expressing the protein in a wet lab.

However, if the AI-designed entity possesses hazardous properties, this evaluation step becomes inherently dangerous. This stands in contrast to evaluating text generated by large language models (LLMs) in the medical or virology domain, where outputs remain directly interpretable by humans and standardized benchmarks exist to assess risks (Chen et al., 2025; Gotting et al., 2025). The inability to safely and reliably assess the biological function of IAB outputs poses a fundamental obstacle to timely risk detection and mitigation. Without robust, trustworthy pLM risk evaluation tools and benchmarks, we risk not knowing the true danger posed by a new IAB or a specific protein design until it has been physically instantiated—potentially too late to prevent harm.

## 5 Open challenges: safeguarding pLMs

On the biosecurity side, traditional regulatory measures are globally insufficient for addressing AI-specific risks. Established frameworks, such as the U.S. Policy on Dual Use Research of Concern (DURC) (U.S. Department of Health and Human Services, Administration for Strategic Preparedness and Response, ASPR), rely on static lists of specific agents and experimental manipulations that fail to capture the versatile nature of tools like pLMs. As such, it does not account for the dual-use potential of IAB (Undheim, 2024). This disconnect extends to other major players: while China's 2020 Biosecurity Law elevates the issue to a national security priority, it remains heavily focused on physical containment rather than algorithmic risks (Min et al., 2025). Meanwhile, in Latin America, governance is hindered by limited institutional awareness and a lack of policy harmonization (Flores-Coronado et al., 2025).

One of the most widely used approaches in biosecurity—**DNA synthesis screening** (SecureDNA, 2025; Baum et al., 2024) aims to prevent the acquisition of matches to regulated pathogens or known hazardous sequences (DiEuliis et al., 2017). Yet red-teaming has exposed severe vulnerabilities: an MIT experiment demonstrated how order splitting and camouflaging allowed synthetic fragments capable of reconstructing the 1918 influenza virus to be purchased from many providers. In that test, 93% of U.S. vendors and 100% of international vendors delivered the disputed fragments. Moreover, a separate adversarial exercise by Microsoft scientists underscored the same risk of evasion (Wittmann et al., 2025). Also, generative models can design entirely novel protein sequences (Dauparas et al., 2022) or potentially generate sequences designed to evade detection (Lu et al., 2023).

**Figure 4:**
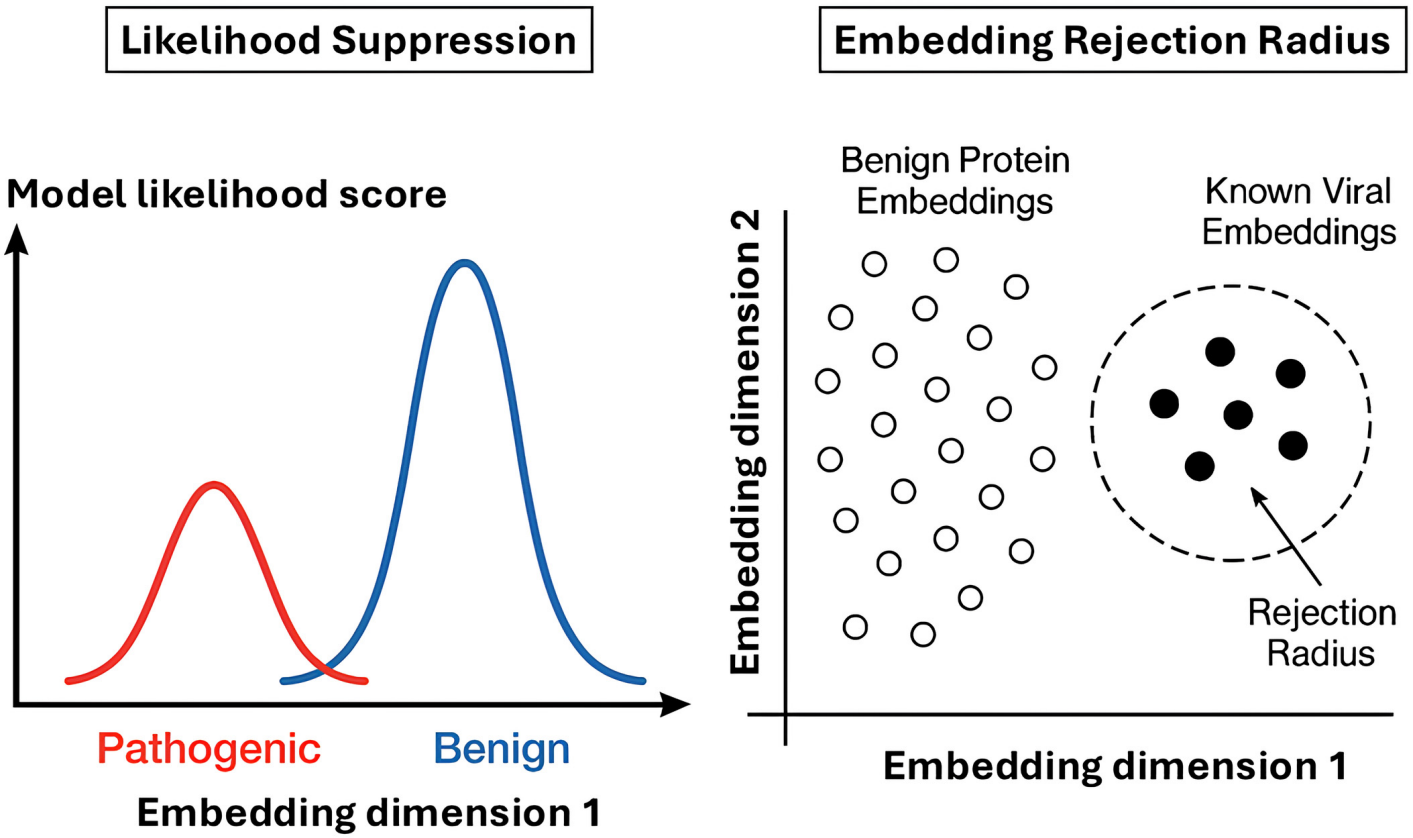
Illustration of examples of training-time and inference-time guardrails for pLMs. Likelihood suppression during training time (Ko et al., 2024) assigns low probability to pathogenic sequences, while an embedding-space rejection radius (Rebedea et al., 2023) blocks generation of sequences too close to known harmful proteins in inference time.

**On the training methodology side, no established safeguard frameworks exist for pLMs**. To address this gap, we explore early-stage technical approaches—adapted from the LLM safety literature—that may help reduce the risk of generating dangerous biological sequences. Broadly, these approaches can be categorized into **training-time guardrails**, which modify the model's learning process to discourage the generation of harmful content; and **inference-time guardrails**, which filter or steer model outputs at the point of generation. One fundamental training-time strategy is *likelihood suppression*, which aims to discourage the model from assigning high probability to harmful sequences (Figure 4). This can be formalized by modifying the training objective to penalize the likelihood of pathogenic sequences:


$$\begin{array}{l} L=L_{\text{original}}-\text{λ}\log P\left(\text{pathogenic}\right) \end{array}$$ (2)

where L represents the likelihood of any sequence and λ controls the strength of the suppression (Ko et al., 2024). However, this approach is not without drawbacks, as safeguards that alter the loss function can be difficult to adopt due to their negative effects on beneficial model uses (Dong et al., 2025). A more adaptive approach to implementing such training-time penalization, or more broadly steering the model toward safer outputs during training, is Reinforcement Learning from Human Feedback (RLHF) (Ouyang et al., 2022; Bai et al., 2022). While no end-to-end implementation of RLHF for pLM safety has been empirically demonstrated, we sketch a conceptual mapping here as a foundation for crucial future research and development in this area. In this context, the pLM acts as a policy generating sequences, while a separate reward model (RM)—potentially trained on datasets of viral protein sequences, structures, and functions—evaluates their potential harmfulness. The pLM can then be fine-tuned using RL algorithms like Proximal Policy Optimization (PPO) (Schulman et al., 2017) to minimize the generation of dangerous sequences. This approach represents an advanced method for instilling safety considerations during the model training phase. Recent work has demonstrated the feasibility of using RL techniques on pLMs for preference optimizations and fine-tuning (Karimi et al., 2024; Stocco et al., 2024; Mistani and Mysore, 2024; Liu et al., 2025; Blalock et al., 2025), suggesting these methods could be adapted for safety purposes. Developing RM for pLM safety could face difficulties, including precisely defining the harmfulness score and obtaining sufficient labeled protein data for it. RLHF for pLMs can inherit issues from LLMs such as reward hacking.

Alternatively, safeguards can be implemented as inference-time guardrails, a strategy most effective for proprietary commercial models where providers embed protections at the API level. In contrast, these external guardrails are less robust for open-source models, as attackers can bypass them by modifying the code directly (Dong et al., 2025).

These methods typically do not alter the underlying model weights but instead apply checks, filters, or steering mechanisms during or after the generation process. This can involve pre-generation constraint conditioning, where generation is guided away from risky regions of the sequence space using techniques like control tokens or latent variable manipulation. A specific example of an inference-time filter is the embedding-space rejection radius (Rebedea et al., 2023) (Figure 4). This method blocks the output of generated sequences whose embeddings are found to be too close to those of known harmful proteins. During inference, a generated sequence's embedding would be compared against a curated database of harmful protein embeddings (e.g., using cosine similarity or Euclidean distance). If a sequence falls within a predefined rejection radius of a known harmful protein, its output is blocked or flagged.

Developing robust and generalizable safeguards, however, will also require standardized benchmarks to evaluate model capabilities in high-risk domains such as viral fitness prediction. To support this, we propose a zero-shot benchmark example (Table 2) built from publicly available viral mutational scanning datasets, which quantify fitness across thousands of viral protein variants. These could enable assessments of whether a pLM can predict viral properties, offering an empirical basis to evaluate dual-use risk, particularly for open-weight models. We acknowledge that the development of such benchmarks might be prone to being misused for designing new viruses; therefore, efforts are needed to widen the evaluation-genration gap–that is, making it harder to generate harmful viruses but easier to detect them. Furthermore, future work should expand on this foundation to develop a more comprehensive dataset.

**Table 2:** Example of a viral fitness dataset for benchmarking pLM viral capabilities.

**Virus**	**Protein**	**Fitness proxy**	**# Variants**
SARS-CoV-2	Spike RBD	Expression score via yeast display (Starr et al., 2020)	~3,800
				Binding affinity to ACE2 (Moulana et al., 2022)	~33,000
Influenza A	Hemagglutinin (HA)	Replication efficiency (Doud and Bloom, 2016)	~10,000
HIV-1	Envelope glycoprotein (Env)	Replication efficiency (Haddox et al., 2018)	~13,000

While our discussion centers on built-in pLM safeguards, we recognize that comprehensive IAB risk mitigation requires a multi-layered defense strategy. We focus primarily on pLM-specific safeguards for several key reasons. First, pLMs represent a critical and currently under-defended chokepoint in the IAB pipeline—while other layers like DNA synthesis screening and laboratory oversight have established (though imperfect) safeguards. Second, pLM safeguards offer broad coverage across multiple downstream applications, potentially preventing harmful sequences from being generated regardless of the specific experimental platform used. However, effective IAB governance requires safeguards across all system components from laboratory-level to model-level.

## 6 Conclusion: from capability to responsibility

Integrating AI, particularly pLMs, with automated experimental biology platforms marks a significant technological leap. The specific biosecurity implications depend critically on the application of the IAB system. For instance, capabilities advanced for exploring small biomolecules or designing novel therapeutic antibodies pose a vastly different and lower relative risk than systems applied to exploring the immune escape of pandemic pathogen variants. The most acute risks emerge when IAB systems are used to rapidly explore complex biological landscapes to optimize high-risk functions, such as viral fitness or immune evasion, in pathogens of concern. Specifically, malicious actors could generate and release multiple variants that escape antibody-based population immunity. Even with intact T-cell immunity, the serial release of such functional, immune-evasive variants could drive repeated global waves of infection and substantial mortality.

Existing AI and biosecurity frameworks fall short of managing these IAB-specific risks. The path forward lies in developing pLM safeguards that can differentiate between these applications—enabling continued innovation in low-risk domains while implementing stringent controls for high-risk uses. Training-time safeguards like likelihood suppression can be calibrated to specifically penalize pathogenic sequences while preserving performance on therapeutic targets. Similarly, inference-time guardrails can implement application-specific screening, blocking outputs in high-risk domains while permitting beneficial uses. Additionally, safety frameworks and safeguards for pLMs should be easy to update, so newly identified weapons-relevant capabilities can be quickly restricted, especially as AI-Bio tools becoming more powerful.

Rather than constraining all IAB development, the goal should be advancing capability selectively while reducing risks differentially. Safeguards could advance on two fronts: (i) the safety of the model itself, by integrating technical controls at the training-time and at inference-time guardrails discussed in Section 5, and (ii) the DNA-synthesis infrastructure where screening must move beyond today's homology-match filters, and be expanded to function-aware or structure-aware methods that use pLMs themselves as screening tools. Unlike LLMs, pLM outputs can be synthesized into real biological threats. Risk must therefore be assessed across the entire pipeline, from design to synthesis, with enforceable safeguards.

## Data Availability

The original contributions presented in the study are included in the article/supplementary material, further inquiries can be directed to the corresponding authors.

## Footnotes

1 *Emerald cloud lab*. Available online at: https://www.emeraldcloudlab.com/ (Accessed April 30, 2025).
